# Interspecific hybridization, polyploidization, and backcross of *Brassica oleracea var. alboglabra* with *B. rapa* var. *purpurea* morphologically recapitulate the evolution of *Brassica* vegetables

**DOI:** 10.1038/srep18618

**Published:** 2016-01-04

**Authors:** Xiaohui Zhang, Tongjin Liu, Xixiang Li, Mengmeng Duan, Jinglei Wang, Yang Qiu, Haiping Wang, Jiangping Song, Di Shen

**Affiliations:** 1 Institute of Vegetables and Flowers, Chinese Academy of Agricultural Sciences; Key Laboratory of Biology and Genetic Improvement of Horticultural Crops, Ministry of Agriculture, Beijing, 100081, China

## Abstract

*Brassica oleracea* and *B. rapa* are two important vegetable crops. Both are composed of dozens of subspecies encompassing hundreds of varieties and cultivars. Synthetic *B. napus* with these two plants has been used extensively as a research model for the investigation of allopolyploid evolution. However, the mechanism underlying the explosive evolution of hundreds of varieties of *B. oleracea* and *B. rapa* within a short period is poorly understood. In the present study, interspecific hybridization between *B. oleracea var. alboglabra* and *B. rapa* var. *purpurea* was performed. The backcross progeny displayed extensive morphological variation, including some individuals that phenocopied subspecies other than their progenitors. Numerous interesting novel phenotypes and mutants were identified among the backcross progeny. The chromosomal recombination between the A and C genomes and the chromosomal asymmetric segregation were revealed using Simple Sequence Repeats (SSR) markers. These findings provide direct evidence in support of the hypothesis that interspecific hybridization and backcrossing have played roles in the evolution of the vast variety of vegetables among these species and suggest that combination of interspecific hybridization and backcrossing may facilitate the development of new mutants and novel phenotypes for both basic research and the breeding of new vegetable crops.

Interspecific hybridization is an important driving force in plant evolution and speciation^1^,^2^,^3^. Following interspecific hybridization, there are two models for the subsequent evolution of plants: genome polyploidization and stabilization, and gene flow with ploidy maintenance^4^,^5^,^6^,^7^. It has been estimated that over 70% of flowering plants have undergone genome polyploidization^2^,^8^. Members of Brassicaceae, a family containing many important vegetable and oil crops, have undergone genomic duplication several times^9^. Crops such as radishes, cabbages, turnips, and Chinese cabbages are diploids. They have undergone ancient hexaploidization and subsequent fractionalization events^10^,^11^,^12^,^13^. The best-known recent genome polyploidization event is the interspecific hybridization of *B. rapa* (A genome), *B. nigra* (B genome), and *B. oleracea* (C genome) to form the allotetraploids *B. juncea* (AB genome), *B. napus* (AC genome), and *B. carinata* (BC genome)^14^. To date, artificial interspecific hybridization and allopolyploids have been primarily synthesized in the laboratory with the goal of exploring the mechanisms underlying polyploidization-dependent evolution and expanding genetic diversity of crops^15^,^16^,^17^,^18^,^19^,^20^,^21^,^22^. However, the evolutionary mechanisms underlying gene flow between homoploids have not been thoroughly investigated.

*Brassica oleracea* and *B. rapa* are two closely related taxa that contain the most important cruciferous vegetable crops. *B. oleracea* is composed of more than 15 subspecies, including *B. oleracea* var. *alboglabra* (Kai-lan, Chinese broccoli), *B. oleracea* var. *acephala* (Acephala, garden cultivars), *B. oleracea* var. *botrytis* (cauliflower and romanesco), *B. oleracea* var. *capitata* (cabbage), *B. oleracea* var. *costata* (tronchuda cabbage), *B. oleracea* var. *gemmifera* (Brussels sprout), *B. oleracea* var. *gongylodes* (kohlrabi), *B. oleracea* var. *italica* (broccoli), *B. oleracea* var. *medullosa* (marrow cabbage), *B. oleracea* var. *oleracea* (wild cabbage), *B. oleracea* var. *palmifolia* (palm cabbage), *B. oleracea* var. *ramosa*, *B. oleracea* var. *sabauda* (Savoy cabbage), *B. oleracea* var. *sabellica* (curly kale), and *B. oleracea* var. *viridis*^23^,^24^,^25^. *B. oleracea var. alboglabra* has been suggested to be a primitive type of kale crop and a possible ancestor of various cultivated *B. oleracea* vegetables^23^,^24^. *B. rapa* contains an even greater number of subspecies and varieties, such *as B. rapa* ssp. *chinensis* (pakchoi), *B. rapa* ssp. *pekinensis* (Chinese cabbage), *B. rapa* var *purpurea* (purple Cai-tai, *B. compestris* L. var *purpurea* was used in the past), *B. rapa* ssp. *parachinensis* (Cai xin), *B. rapa* ssp. *dichotoma, B. rapa* ssp. *japonica, B. rapa* ssp. *Narinosa* (wutacai), *B. rapa* ssp. *Nipposinica* (mizuna), *B. rapa* ssp. *oleifera, B. rapa* ssp. *rapa* (turnip), and *B. rapa* ssp. *trilocularis*^23^,^24^,^26^. The oldest members of *B. rapa* were planted thousands of years ago. However, most varieties of *B. compestris* cultivars, the hundreds of landraces of pakchoi in southern China and Chinese cabbage in northern China, emerged between the 14^th^ and 17^th^ centuries^27^. Though this evolutionary boom has attracted much attention, the mechanisms underlying it remain unclear.

In the present study, an interspecific hybrid of *B. oleracea* var. *alboglabra* and *B. rapa* var. *purpurea* (the primitive type of cultivated *B. oleracea* and *B. rapa*) was generated. After two rounds of backcrossing of the synthetic allotetraploid plant with *B. rapa* L. var. *purpurea*, the morphological variation and genetic composition of the progeny were investigated. The stereotypical traits of *B. oleracea* var. *gemmifera* (Brussels sprouts), *B. oleracea* var. *gongylodes* (kohlrabi), *B. chinensis* (pakchoi), and *B. pekinensis* (Chinese cabbage) were phenocopied or partially phenocopied in the BC_2_ plants, providing direct evidence in support of the hypothesis that interspecific hybridization and backcrossing played roles in the evolutionary expansion of vegetable varieties in *B. oleracea* and *B. rapa*. The intergenomic recombination and asymmetric segregation of chromosomes could be one of the principal mechanisms underlying this process. Many interesting mutants and previously unknown phenotypes were obtained, indicating that the combination of interspecific hybridization and backcrossing may facilitate the development of more mutants and novel phenotypes.

## Results

### Creation of hybrid plants by embryo rescue

**Figure 1:**
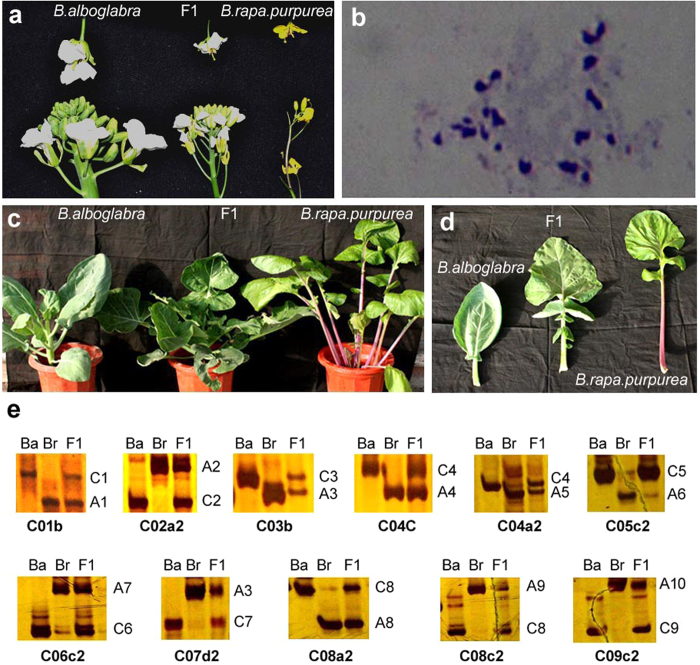
The F1 hybrid of *B. oleracea* var. *alboglabra* and *B. rapa* L. var. *purpurea*. (**a**) Inflorescences and flowers; (**b**) Chromosome microscopy of root tip cells from the F1 hybrid plant, n = 19; (**c**) Plants at the vegetative growth stage; (**d**) Rosette leaves. (**e**) Chromosome specific SSR analysis of F1 plant, Ba, *B. oleracea* var. *alboglabra*; Br*, B. rapa* L. var. *purpurea*; C1–C9 and A1–A10 labeled on right side of lanes indicat corresponding chromosomes, bold alphanumeric characters below each gel indicate the SSR markers. Photographs were taken by Zhang X. and Li X.

A cytoplasmic male sterile line of *B. oleracea* var. *alboglabra* was hand-pollinated with pollen from an inbred line of *B. rapa* L. var. *purpurea*. On the 8^th^ day after pollination, the young siliques were harvested, the surfaces were sterilized, and then ovules were dissected out and grown in embryo culture media. Three out of the ≈400 ovules developed into seedlings. Morphologically, the hybrid plants fall between their parents (Fig. 1). Chromosome analysis from the root tip cells revealed that the hybrid somatic cells contained 19 chromosomes (Fig. 1b). A series of codominant Simple Sequence Repeats (SSR) markers indicated ten of which were derived from *B. rapa* L. var. *purpurea*, and nine of which were derived from *B. oleracea* var. *alboglabra* (Fig. 1e). The hybrid plants were male sterile and produced no pollen. On the main stalk, the embryos were lethal when hand-pollinated with pollen from either *B. rapa* L. var. *purpurea* or a fertile *B. oleracea* var. *alboglabra* plant. However, some adventitious stalks generated from the bases of the plants produced several seeds by backcrossing with *B. rapa* L. var. *purpurea*. These partially fertile stalks were stout with shortened internodes that distinguished them morphologically from the original plants, indicating that these branches may underwent a spontaneous chromosomal doubling.

### Morphological divergence among BC_1_ (AAC) plants

**Figure 2:**
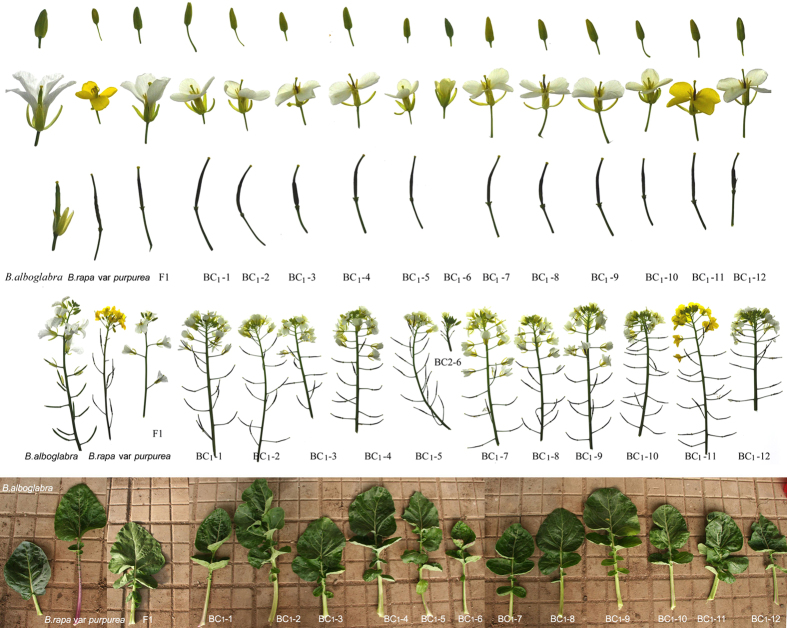
Morphological variations in BC_1_ plants. Panels from top to bottom show buds, flowers, young siliques, inflorescences, and rosette leaves. Photographs were taken by Zhang X.

A total of 72 seeds were harvested from the partially fertile F1 stalks that were hand pollinated with *B. rapa* var. *purpurea*. After sowing, 67 seedlings germinated and completed a life cycle. Within this population, morphological variations were observed in the size and shape of the leaves, the size and color of flowers and stalks, the structure of the inflorescence and the presence of surface wax (Fig. 2). White petals and surface wax were the dominant traits controlled by the genes from C genome, as indicated by the F1 plants (Figs 1 and 2). The occurrence of yellow petals and waxless surfaces suggests that some of the chromosome segments from the C genome had been lost from the BC_1_ plants.

### Morphological traits of BC_2_ progeny

**Figure 3:**
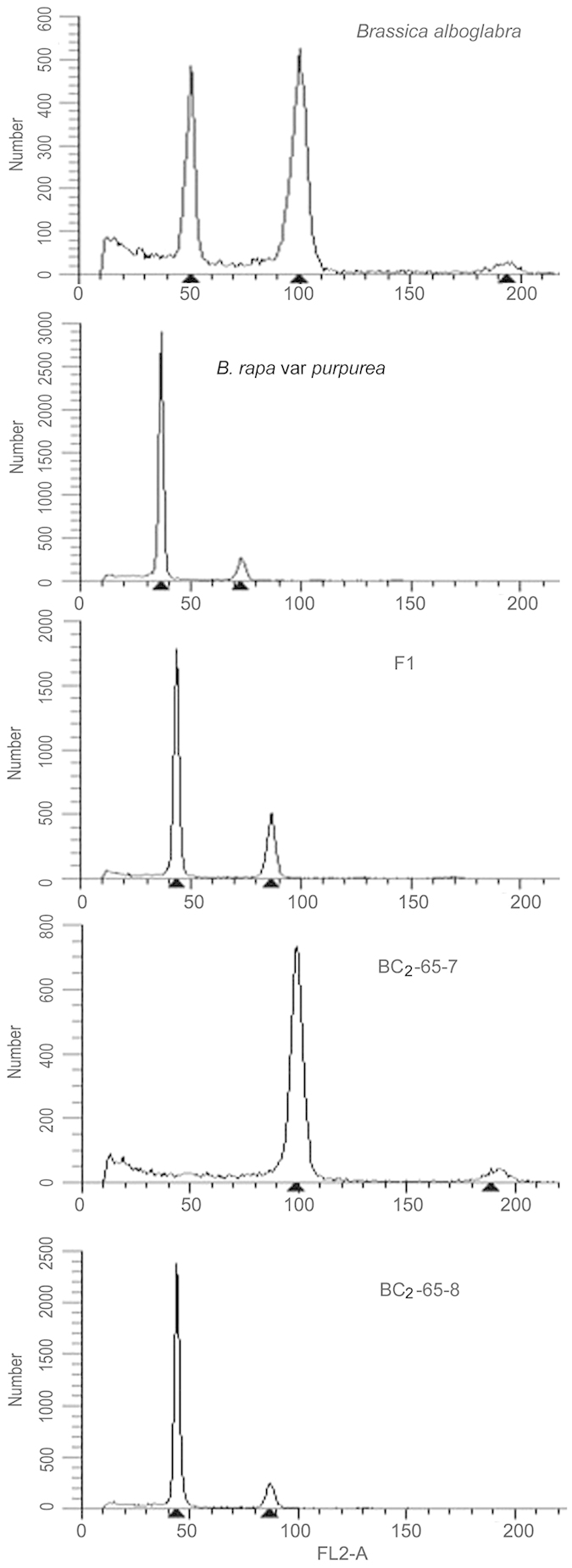
Flow cytometry analysis of parents and progeny. Leaf tissue cells were analyzed. The F1 plant contains chromatin content with the mean value of its parents. BC_2_-65-7 and BC_2_-65-8 are BC_2_ individuals showing significant differences in chromatin content.

**Figure 4:**
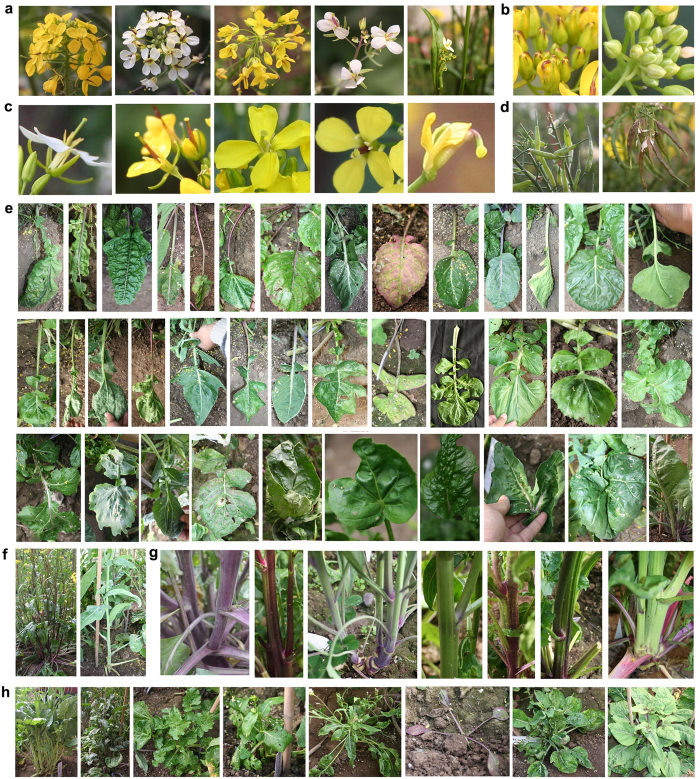
Morphology of BC_2_ plants. (**a**) Inflorescences vary in structure and color. (**b**) Purple or white patches on the tips of buds. (**c**) The pistils vary in color, length, and shape. (**d**) Colors of the siliques. (**e**) Differences in rosette leaves. (**f**) Poly and monostalks. (**g**) Differences in stalk in color and waxiness. (**h**) Variations in plant architecture. Photographs were taken by Zhang X.

**Figure 5:**
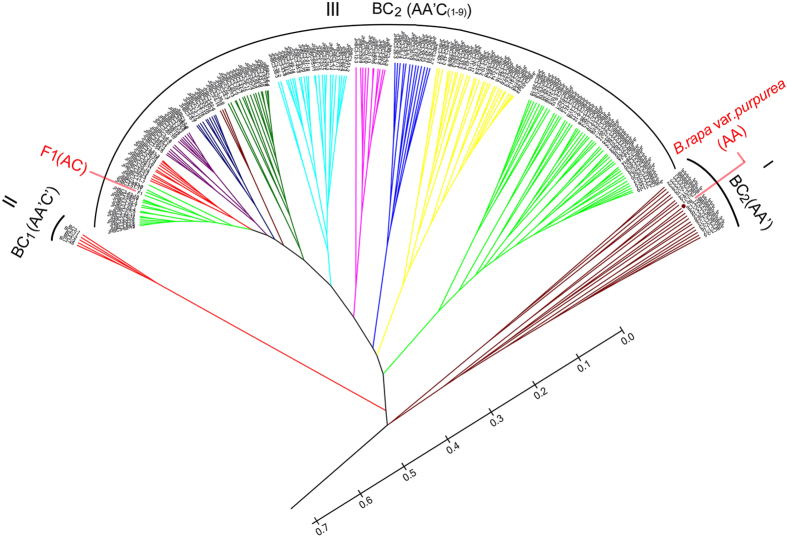
Clustering of two generations of backcrossed progeny and their ancestor plants. The UPGMA tree was constructed using MEGA 5 and morphological traits.

After hand pollination with *B. rapa* L. var. *purpurea* as the male parent, all of the 67 resulting BC_1_ plants were fertile. These 67 BC_2_ lines produced 935 seedlings. It was deduced that each plant in this generation contained a genome composed of AA′C_(0–9)_ (A′ probably contains segments from C due to chromosomal recombination; C_(0–9)_ contains 0–9 chromosomes from the C genome that probably contain segments from A). Flow cytometry showed that the chromatin content diverged significantly (Fig. 3). Morphologically, the BC_2_ plants bore a stronger resemblance to *B. rapa* L. var. *purpurea*, but the detailed traits diverged significantly among individuals within the population (Fig. 4). The plants were clustered based on 17 morphological traits, including plant height, architecture, stooling, stalk shape, stalk color, waxiness of the stalk, brawniness of the stalk, lateral stalk number, rosette leaf shape, rosette leaf color, rosette leaf crimp, waxiness of the leaf, petiole color, bolting time, flowering time, petal color and style structure. As shown in Fig. 5, 19 of the 274 (6.93%) BC_2_ plants clustered with *B. rapa* L. var. *purpurea* (group I), indicating that these plants were of the AA′ genotype, from which the chromosomes derived from the C genome had been eliminated. However, the individuals within this group were found to differ from each other significantly, indicating that recombination between chromosomes from the A and C genome occurred frequently during two rounds of meiosis, resulting in the replacement of many segments of the A′ genome with fragments from the C genome. Group II comprised BC_1_ plants. These plants were less diverse morphologically because they underwent only one round of recombination and contained an AA′C′ genome. The other plants were clustered according to nine major terms in group III, indicating the wide range of segregation events and complicated genetic interactions.

### Phenocopying of *Brassica* vegetables by BC_2_ progeny

**Figure 6:**
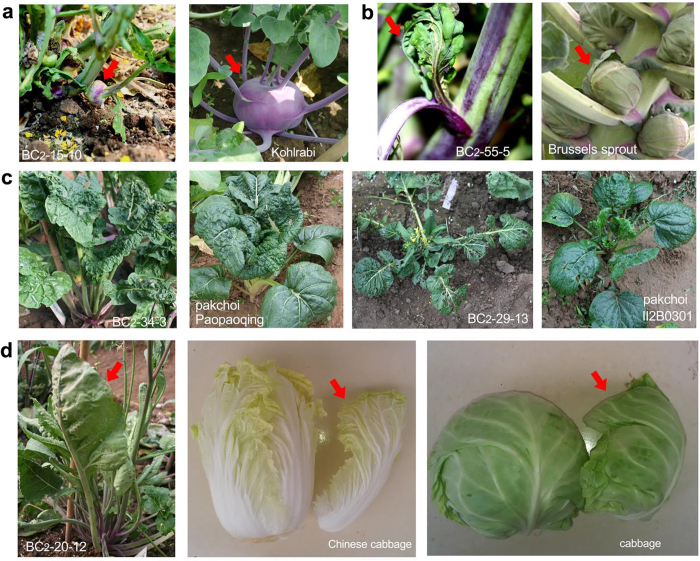
Phenocopying of other *Brassica* vegetables by BC_2_ progeny. (**a**) A BC_2_ plant (BC_2_-15-10) generated enlarged bulbs (red arrow) that resemble kohlrabi. (**b**) A BC_2_ plant (BC_2_-55-5) produced axillary bud balls (red arrow) characteristic of Brussels sprouts. (**c**) BC_2_ plants (BC_2_-34-3 and BC_2_-29-13) with a phenotype nearly identical to landraces of pakchoi. (**d**) A BC_2_ plant (BC_2_-20-12) produced leaves that folded inward (red arrow), a fundamental trait for developing the leafy head found on Chinese cabbages and other cabbages. Photographs were taken by Zhang X. and Li X.

Some BC_2_ plants displayed traits characteristic of vegetables produced by plants other than their progenitors. As shown in Fig. 6, one BC_2_ plant generated enlarged corms that resemble kohlrabi (*B*. *oleracea* var. *carlorapa*) (Fig. 6a). Some BC_2_ plants produced axillary bud balls, which are characteristic of Brussels sprouts (*B. oleracea* L. var. *gemmifera*) (Fig. 6b). A number of BC_2_ plants displayed a phenotype nearly identical to several pakchoi landraces (*B. rapa* ssp. *chinensis*). For example, some BC_2_ plants phenocopied the “Paopaoqing,” a pakchoi landrace that produces characteristic dark green wrinkled rosette leaves (Fig. 6c). The leaves of the BC_2_ plants frequently folded inward (Fig. 6d), a fundamental developmental process that leads to the formation of the leafy head in cabbage and Chinese cabbage. This suggests that the leafy head formation if the petiole will be short and that the inner leaves will fall inward. These phenotypes morphologically recapitulate the evolutionary history of several *Brassica* vegetable varieties.

### Interesting mutants and novel phenotypes

**Figure 7:**
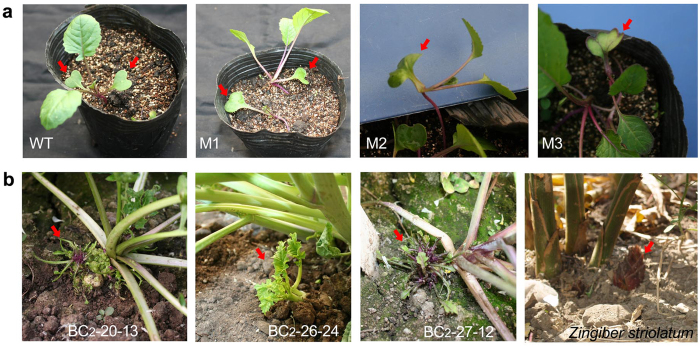
Mutants generated from BC_2_ plants. (**a**) WT, wild-type normal plant; M1, M2, and M3, monocotyledon mutants; M3 clearly shows a monocotyledon formed by this combination. (**b**) The mutants (left three panels) generated young plants from their underground root systems that resemble *Zingiber striolatum* Diels. Photographs were taken by Zhang X.

In addition to the evidence presented for morphological variation and evolutionary recurrence, many interesting mutants were generated at a relatively high frequency. For example, one mutant presented two cotyledons that merged into a monocotyledon (Fig. 7a). The backcross progeny segregated into mono and dicotyledonous individuals at a 1:1 ratio, indicating that the mutation is controlled by a single dominant gene. This mutant might be a good model for the study of cotyledon development and determination of the molecular basis of mono and dicotyledonous segregation, which is a fundamental question in the evolution and phylogeny of flowering plants. Another interesting mutant generated young plants from its underground root system (Fig. 7b). Similar schedules have been adopted as a primary method of reproduction by many plants. This mutant might be useful for studying mechanisms of this kind. Plants resembling bamboo shoots and *Zingiber striolatum* could be used as new vegetable cultivars.

### Intergenomic recombination and asymmetric segregation as indicated by SSR analysis

**Figure 8:**
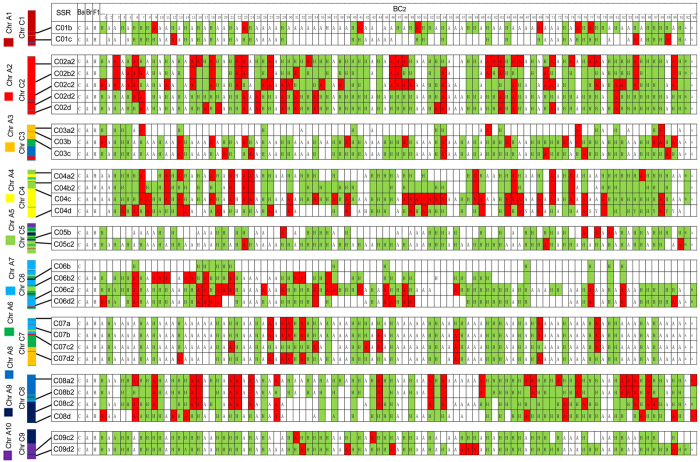
Chromosome recombination and segregation as indicated by SSR markers. The C chromosomes are shown at the left. The segments homologous to A01–A10 chromosomes are indicated with ten different colors. The chromosome loci of the SSRs are given as solid lines. C, A, and H indicate the C chromosomes, A chromosomes, and hybrid type bands. The detection of C chromosome was highlighted in green, the loss of C chromosome segments resulted from intergenomic recombination are highlighted in red.

**Figure 9:**
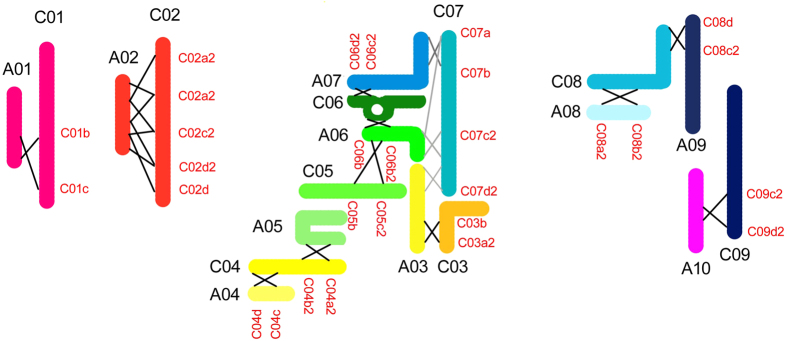
Homeologous chromosome recombination model. A sketch of homeologous chromosome recombination between the A and C genomes constructed with the events detected by SSR analysis. A01-A10, *B.rapa* chromosomes. C01-C09, *B. oleracea* chromosomes. Red alphanumeric characters indicate the SSR markers. Cross lines indicate the recombination events.

To investigate the genetic mechanism underlying the aforementioned morphological observations, 30 SSR markers (2–5 per chromosome, details in Table 1) were designed and used for genetic analysis of 93 BC_2_ plants, the F_1_, and their initial progenitors. As shown in Fig. 8, the BC_2_ plants contained 0–9 whole or partial C chromosomes from *B. oleracea* var. *alboglabra*. Intergenomic recombination between A and C chromosomes was detected between each pair of adjacent markers. Because both A and C genomes have been extensively rearranged since their divergence, the homeologous recombinations can result in asymmetric segregations with chromosome deletion and duplication, as demonstrated in Fig. 9.

**Figure 10:**
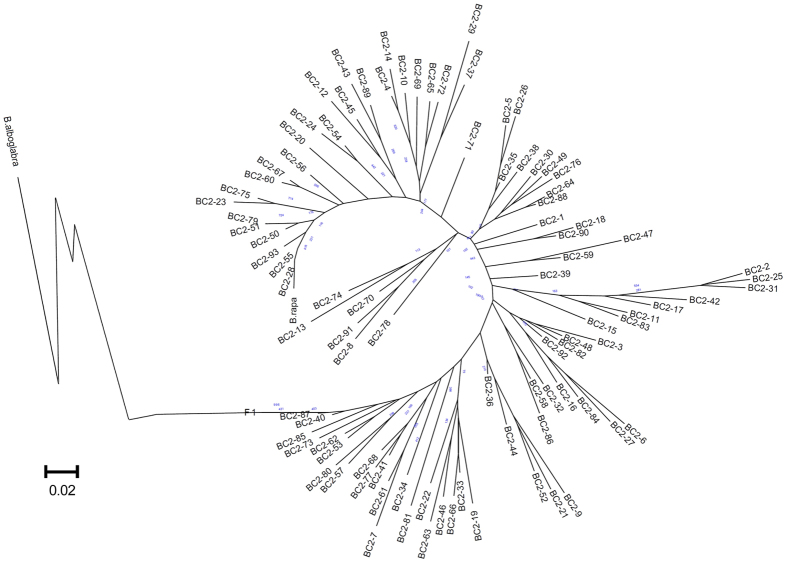
Neighbor-joining tree. The tree was constructed with 30 SSR markers using MEGA 5. The blue numbers indicate the bootstraps.

A neighbor-joining (NJ) tree was generated based on this set of SSR data (Fig. 10). The BC_2_ plants also clustered in nine major branches, which are in accordance with the tree constructed with morphological traits (Fig. 5). Due to two generations of backcrossing with *B. rapa* L. var. *purpurea*, it is not surprising that the *B. oleracea* var. *alboglabra* outbranched far from the BC_2_ groups. The F_1_ and *B. rapa* L. var. *purpurea* clustered separately in two outside branches, which were again consistent with the morphological tree. One of these two branches contained the BC_2_ plants harboring nearly the whole set of C chromosomes and the other those that had lost nearly all of the C chromosomes. The inner branches were composed of those harboring many different numbers of C chromosomes. These molecular data confirmed the morphological observations.

### InDel mutations detected in BC_2_ plants

**Figure 11:**
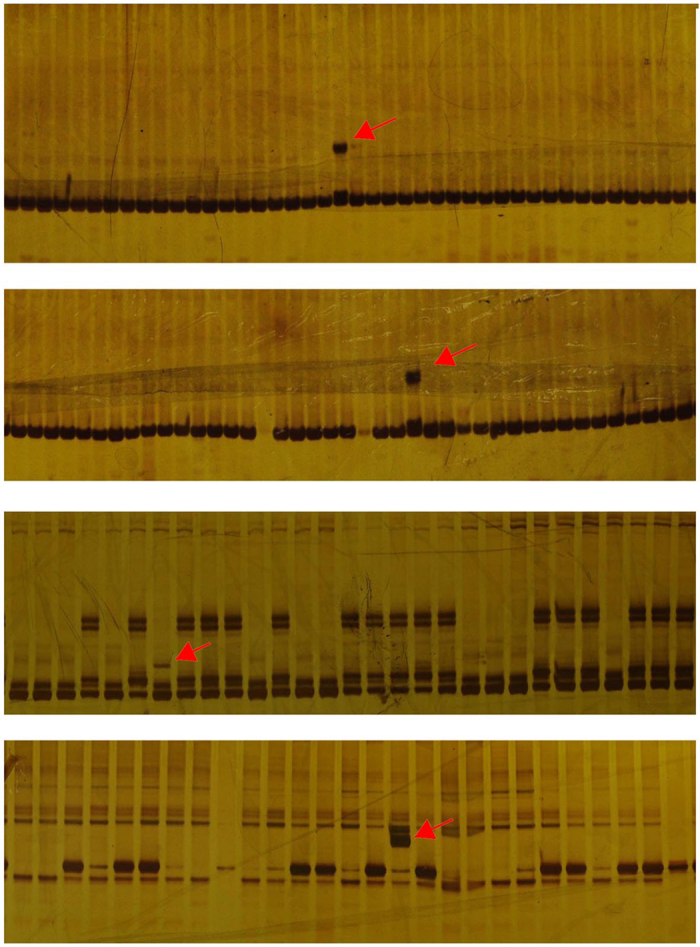
InDel mutations detected by PCR. The red arrows point the mutational bands.

The SSR analysis also detected four novel bands in the BC_2_ plants (Fig. 11). All of these four mutations are short stretch insertions. The mutation rate was 0.15%, much higher than the spontaneous mutation rate, indicating the minor genomic alteration such as short stretch insertion was induced by interspecific hybridization.

## Discussion

Many interspecific hybrids have been produced from various types of wild and cultivated A and C genome plants^15^,^16^,^17^,^18^,^19^,^20^,^21^,^22^. However, this is the first report of the hybridization of the two flower stalk vegetables, *B. oleracea* var. *alboglabra* and *B. rapa* L. var. *purpurea*. Morphological analysis, chromosome counting, flow cytometry and SSR analysis demonstrated that hybridization was successful. The hybrid plants showed a relatively high degree of spontaneous chromosome doubling. Because the initial progenitors were highly homologous, the morphological divergence among the BC_1_ plants could not have been derived from genetic segregation between sister chromosomes. Mechanisms such as recombination between homoeologous chromosomes and alterations in the expression pattern and DNA methylation status of genes have been proven to lead to rapid phenotypic changes in newly synthesized allopolyploids^16^,^17^,^18^,^19^,^20^,^21^,^22^,^28^,^29^,^30^,^31^. The DNA methylation and expression level were not analyzed in these newly synthesized hybrids. However, homoeologous chromosome recombination was found to be active in this system. The changes in qualitative traits, such as flower color, indicate the non-homologous chromosomal recombination and gene loss took place during meiosis in the F_1_ plants^32^,^33^. The relatively low fertility rate of the first round of backcrosses indicated that non-homologous chromosomal recombination occurred extensively, resulting in female gamete lethality due to chromosome disorder during meiosis. This is consistent with the observation that newly synthesized allopolyploids undergo consistent chromosome crossover and recombination^17^,^34^,^35^.

Chromosome recombination requires homologous sequences for DNA pairing. Sequencing of cabbages and Chinese cabbages has shown that the A and C genomes share many homologous blocks^10^,^13^, presenting opportunities for recombination. Both the A and C genomes underwent two rounds of genome duplication and produced three subgenomes^9^,^36^. This type of genome redundancy provides considerable plasticity for non-homologous recombination between A and C chromosomes. These facts may be the reason for the extensive homeologous recombination displayed in the hybrid plants, which leaded to chromosome deletion, duplication and rearrangement in high frequency and finally resulted in the numerous phenotypes observed in the BC_2_ progenies. On the other hand, our backcrossing strategy also contributing to the high frequency homeologous recombination observed, because a complete single set of A chromosomes always obtained from the recurrent parent, which will rescue the lethal chromosome rearrangements in the maternal plants.

One well-known example of interspecific hybrid evolution is the U’s triangle model, in which three tetraploids, *B. napus*, *B. juncea*, and *B. carinata*, were proposed to have evolved from hybrids of any two of the three diploids *B. rapa*, *B. nigra*, and *B. oleracea*^14^. Each of the three major *Brassica* vegetable species, *B. rapa*, *B. oleracea*, and *B. juncea*, have a large number of subspecies and varieties and various crop cultivars that produce a diverse variety of edible organs. Previous studies have suggested that these plants were domesticated independently in China, Europe, and the Middle East^37^,^38^,^39^. Primitive types, such as kale and turnip, may have been cultivated more than 5,000 years. However, historical records indicate that most other types emerged suddenly between 800–400 years before present^27^. The evolutionary history of the expansion of this cultivar is still a mystery; the natural variation and selection model cannot explain this rapid speciation. Recent re-sequencing and comparison between three subspecies of *B. apa*, a turnip, a rapid cycling, and the reference genome of Chinese cabbage estimated the date of divergence among the three morphotypes at approximately 250,000 YA, long predating the date of domestication^40^. One explanation for this paradox is that the genetic variation may have come from interspecific hybridization. The present study provides solid evidence that interspecific hybridization and backcrossing have the potential to generate multiple crop types within the *B. rapa* and *B. oleracea* clans. It is here proposed that the primitive crop types of *B. rapa* and *B. oleracea* were domesticated independently in China and Europe for more than two thousand years and evolved slowly and smoothly until the 12–14^th^ centuries, when the crops were introduced to each other between China and the Mediterranean via the Maritime Silk Road. These interspecific crossings were spontaneous and occurred in quotidian settings such as kitchen gardens. Backcrossing to *B. rapa* and *B. oleracea* crops took place in China and the Mediterranean, respectively, due to the population proportion effect. Genetic rearrangement and morphological variation manifested in the progeny of these crosses. Then, various traits were selected according to the selector’s preferences and were consequently refined and stabilized in later generations to form the diverse varieties and cultivars known today. This scenario is also supported by historical biogeographical data that all of the varieties of *B. rapa* were first generated in southern China, where only one Chinese native kale crop, *B. oleracea* var. *alboglabra*, coexists with pakchoi cultivars, including *B. rapa L.* var. *purpurea*^27^,^41^. Even the Chinese cabbage, which is currently planted through much of northern China, Korea, and Japan, originated in southern China and was later distributed elsewhere^27^. In the current model, it was not possible confirm that the two plants used in this study were the initial ancestors of current cultivars, but a trend toward a polychronism was observed, suggesting that multiple interspecific hybridization events took place and that many *Brassica* plants, including allotetraploid crops, could have participated in them.

Mutants are important resources for genetic research and for plant breeding. Natural mutation is rare and the mutants are difficult to obtain. Several methods, including EMS and γ-radiation, have been used to induce mutations^42^. The majority of these methods induce point mutations, creating loss-of-function mutants. The results of the present work showed distant crossing to be a powerful method for producing mutations, including valuable gain-of-function mutants. In distant hybrids, mutants can be generated by chromosome fragment deletion and gene conjunction, which is called “genome reshuffling,” and by activated transposable elements, which induce mutations termed “genome shock”^16^,^17^,^43^. It is not easy to use linkage-map-based cloning on these types of mutants, so they have not been widely used for functional genetic analysis. Currently, with the rapid development of sequencing technology and the accompanying dramatic reduction in cost, these mutants may be more frequently utilized in genetic studies.

Beside the large chromosome fragment rearrangements, minor genomic alteration such as short stretch elimination and insertion also play important roles in the evolution of interspecific hybrid plants^30^. Though only four InDel mutations have been detected in this study, it is far from reflecting the true mutation level in these plants. Because SSR is not an effective mutation detecting tool and it cannot detect the point mutations.

The use of distant cross technology in the plant breeding industry have a long and glorious history. Many important agronomic traits, such as male sterility, disease resistance, and stress tolerance have been introduced to cultivars from wild and interspecific relatives by distant crossing^44^,^45^,^46^,^47^,^48^. However, crop diversity is currently in sharp decline, and current vegetable innovation cannot match that of the pre-industrial age. One reason for this trend is that commercial seeds are highly homogeneous; thus, new traits are not likely to occur nor to be adopted to form the new varieties. The present study shows that artificial distant hybridization and backcrossing can generate new traits at a relatively high frequency. Some of the new traits have the potential to breed new vegetable cultivars. For this reason, it is here proposed that breeders use distant hybridization and backcrossing strategies to generate new types of crops for the enrichment of the human diet rather than limiting themselves to the transfer of existing traits from one plant to another for the minor modification of commercial cultivars.

Among the BC_3_ plants, typical traits such as enlarged corms, bud balls, wrinkled rosette leaves, leaf folding, mono-cotyledon, and root-born seedlings were also observed, indicating that these traits are heritable rather than epigenetic or due to phenotypic plasticity. However, because the maternal progenitor *B. oleracea* var. *alboglabra* is a cytoplasmic male sterile line, their progeny cannot self, so the traits cannot be reproduced stably. After generations of backcrossing, the traits are gradually lost and the phenotype finally approaches that of the recurrent parent *B. rapa* L. var. *purpurea.* This study is to be repeated using a fertile system, and further stabilize and manifest the traits by selfing and sister crossing, which will provide stronger support for the hypothesis raised in this study.

## Material and Methods

### Plant material and growth conditions

*B. oleracea* var. *alboglabra* 4E286 is a cytoplasmic male sterile line. The *B. rapa* L. var. *purpurea* HCT3 is an inbred line. The seeds of these plants and their progeny were surface-sterilized in 1% NaClO and germinated on glass Petri dishes at 25 °C. After germination, seedlings were transplanted into a mixture of peat soil (peat:moss:perlite:vermiculite soil = 3:2:1:1). The seedlings were transplanted to a greenhouse after 2 weeks and were watered and fertilized regularly.

### Distant hybridization and embryo rescue

The *B. oleracea* var. *alboglabra* cytoplasmic male sterile line 4E286 served as the maternal parent. The *B. rapa*L. var. *purpurea* inbred line HCT3 served as the paternal parent. The inflorescence was bagged and hand-pollinated one day later. Eight days after pollination, the siliques were harvested and surface-sterilized in 70% ethanol for 30 s followed by 1% NaClO for 15 min and washed three times in purified, sterile water. The siliques were dissected under a stereomicroscope on a superclean bench. The embryos were cultivated in a solidified MS medium containing 2 mg/L 6-benzyladenine (6-BA) and 0.2 mg/L 1-naphthaleneacetic acid (NAA) and then placed in a tissue culture room at 25 °C with a 16:8 light-dark cycle^49^. The germinated seedlings were propagated on the same medium and then transplanted to a root-induction medium composed of solidified MS + 0.2 mg/L NAA. The rooted plants were transplanted to soil, kept humid for a week, and then cultivated in a greenhouse.

### Morphological observation and cluster analysis

The morphological traits recorded include shape, color, the crimp and wax of the cotyledons, rosette-leaves, stem-leaves, petioles, stalks, flowers, and siliques. The plant height, architecture, bolting time, stooling and brawniness of stalk, number of lateral stalks, and flowering time were observed and measured at the seedling, vegetative, bolting, flowering, and fruiting stages. The morphological data were used for cluster analysis, and the UPGMA tree was constructed using MEGA 5^50^.

### Chromosome observation

Root tips (1 mm) were cut and incubated in 2 mM 8-hydroxyquinoline for 4 h. The tissues were fixed in Carnoy’s Fluid (3:1 ethanol:acetic acid, v/v) for 24 h, then stored in 70% ethanol at 4 °C. The tips were digested in 1 M HCl at 60 °C for 10 min and washed twice in distilled water. Then the apical meristems (i.e., the white spots at the tips) were sliced and squashed in a drop of 10% Carbol fuchsin. The chromosome spreads were observed under a microscope.

### Flow cytometry analysis

Rosette leaves (1 cm^2^) were diced with a razor blade in 1 mL of Galbraith’s buffer (45 mM MgCl_2_, 30 mM sodium citrate, 20 mM 3-(N-morpholino) propanesulfonic acid [MOPS], 0.1% (v/v) Triton X, pH = 7)^51^. Then the diced leaves were mixed with another 1 mL of Galbraith’s buffer and filtered with a nylon mesh (50 μm aperture). Cells were harvested from the solution in a centrifuge at 1000 rpm for 5 min, then suspended in 200 mL of 50 mg/L propidium iodide (PI) and incubated in the dark for 20 min. The DNA content was assayed on a flow cytometer (BD FACSCalibur, NJ, U.S.) with excitation at 488 nm.

### SSR analysis

The SSR markers were designed based on comparison between the genomes of *B. oleracea*^10^ and *B. rapa*^13^. The genome sequences were downloaded from (ftp://bradata:zhl410ivf@brassicadb.org/Brassica_oleracea/Bol_Chromosome_V1.1/BOL.seq.lst.new.chr20110802_check.fa.gz) and (ftp://bradata:zhl410ivf@brassicadb.org/Brassica_rapa/Bra_Chromosome_V1.5/Brapa_sequence_v1.5.fa.gz). The SSRs were firstly identified in *B. rapa* genome using MIcroSAtellite identification tool-MISA (http://pgrc.ipk-gatersleben.de/misa/). Then the primers were design on primer 3^52^. Subsequently, the primers were mapped on *B. oleracea* and *B. rapa* genome using Electronic PCR (e-PCR)^53^. The SSR primers which produced fragments of more than six nucleotides difference between the two plants and also have only one local in each genome were retained. From which, 66 primer pairs equidistributed on the A genome were manual selected and experimentally tested via PCR analysis on *B. oleracea* var. *alboglabra* and *B. rapa* L. var. *purpurea*. The 30 SSR primers (Table 1) generate clear distinguishable bands were used for further analysis. Genomic DNA was extracted from leaf tissues using the cetyl trimethylammonium bromide method. PCR amplifications were performed in a 20 μL volume containing 20–50 ng template DNA, 0.5 pmol primers, 0.5 U Taq enzyme and 1× PCR reaction buffer. Reactions were performed with an initial denaturation step of 3 min at 95 °C; followed by 35 cycles of 95 °C for 30 s, 56 °C for 30 s, and 72 °C for 30 s; and a final extension at 72 °C for 10 min. The PCR products were then separated on an 8% polyacrylamide gel electrophoresis and visualized with silver staining.

## Additional Information

**How to cite this article**: Zhang, X. *et al.* Interspecific hybridization, polyploidization, and backcross of *Brassica oleracea* var. *alboglabra* with *B. rapa* var. *purpurea* morphologically recapitulate the evolution of *Brassica* vegetables. *Sci. Rep.* **6**, 18618; doi: 10.1038/srep18618 (2016).

## Figures and Tables

**Table 1:** Characteristics of the 30 analyzed simple sequence repeat (SSR) markers.

SSR	primer-F	primer-R	Band in A genome (bp)	Band in C genome (bp)	Locus on A genome	Locus on C genome
C01b	GCAACACTTGCTGTTTTGGA	GCTGAGGTAGGAAGGGAAGG	207	233	A01_15917060	C01_20708918
C01c	ATCAACGAGGATGTTGGAGA	TGACCCAATCAGGTCAAGAA	189	183	A01_20992040	C01_31003906
C02a2	GATCGACCTTCCATCACGTC	CCTTGTGCAAGAAGGTGTTG	199	176	A02_4660427	C02_6784924
C02b2	ATATACACATGGCCGGTGCT	TTGGTGGAGTTTCCGAGTTT	114	100	A02_11831311	C02_16112719
C02c2	TTGGCTTCAACGTGTCTGAG	CCACTTAGGCGATGGTGTTT	179	146	A02_15538343	C02_24870903
C02d2	TGGGTTCGAATAAGTCGGAT	CTTGGCCCATCATCAAAACT	278	193	A02_21212404	C02_37480057
C02d	GTCAGGGCCGGTTGGTAT	CCAAAACCGAACTAAACGGA	278	266	A02_26805121	C02_42253466
C03a2	GGACTAGGCCCAGAAAGGTC	ATGAGAATCACACGCGTCAC	181	141	A03_5096072	C03_5782559
C03b	CCGGACCATAAATTATCGCA	GCTCCTCCTCCTCCATCTTC	165	174	A03_9810560	C03_11586692
C03c	AGCCGTTGAATCATAATGGC	AATGACAAACGCCGGTCTAC	142	100	A06_16840545	C03_29211252
C04a2	AAAGTCAGGCGTCTTTCTGC	TTGTTGTTGTATAGACCAGACA	269	137	A05_3393634	C04_6311603
C04b2	CATCACCACCATCTACACGC	ATACAAACTGCCGAGACGCT	153	130	A05_7742417	C04_17130510
C04c	GCAAAGACCTTTTTGCAAGC	ACCGACCAAAGGTGAACAAC	213	253	A04_9337281	C04_28980301
C04d	CGAAGAAATGGGCAATGAGT	CCATGGTCGGACAATCTCTT	123	93	A04_18399294	C04_40018307
C05b	CTCGCATTGAAGGAGTGTCA	ATCTCCAGCCTTTTCCAGGT	263	255	A06_6560520	C05_13746011
C05c2	AAAAGCAACCAAAGCGCTAA	TCGTTTCCTTGATTGCTTCC	139	114	A06_8543638	C05_21938996
C06b	CGTTGTTTGTCGTCTGCATC	GCCGAATCAGAACCGTTAGA	246	238	A06_1811353	C06_13619390
C06b2	TGGAATCACTTCAGCGTTCA	ACGAGTTTTCTCGGCTTTCA	265	72	A06_14493472	C06_17947219
C06c2	TCAGTATTCTCATCGCCCAA	AGGTCGACCAAAGACGAAAA	188	146	A07_15806647	C06_28866205
C06d2	GCTTCATTGGATCCCACATC	GGGTTCGTGATTGATGGTAAA	143	117	A07_19411829	C06_35244684
C07a	ATCCGACCAGTAACGTCCAG	GGGGGATTACCTTCATCTTTT	217	208	A06_24653900	C07_463379
C07b	CCCGTAGTTAGAACACCCGA	GCGCAGACCTAGCCTTATTG	248	265	A07_8821086	C07_16930026
C07c2	TGATGGGTAAAATGCTCCAA	CTGCGTCTTTTCTTCCAGTG	160	125	A06_23763438	C07_27826196
C07d2	CGCGTAGAAGTAGTTCCAGTG	TCATCAAACGTGGAGGTCAA	167	131	A03_23672505	C07_41422404
C08a2	TGTTTTCAGCACCTTTTCAAGA	CAAGCAGTGCAACCACAAAG	241	129	A08_5763859	C08_5598895
C08b2	TGCTGCTAAGTCTAGTCCACAA	CCTCAAGATCCACAATGCCT	248	178	A08_19250863	C08_18384496
C08c2	ACTCTCAACAATGGCAAGGG	GCTGTTTTCCATATTCGGCT	215	159	A09_31431992	C08_32596668
C08d	CATCGGCCGTAACAAAGAAT	CAGAAACGGGATTCGATCAT	253	231	A09_38876771	C08_41482344
C09c2	TAGCCAACTAACATGGCACG	GCCCACATCTTTCAAAACAAA	177	143	A10_9735500	C09_28720170
C09d2	AAGCTACTTGCCTCACTATCCT	TGATTTCACATAAGGTCCCCA	250	159	A10_12909906	C09_35598537
